# Processing Connectives with a Complex Form-Function Mapping in L2: The Case of French “En Effet”

**DOI:** 10.3389/fpsyg.2017.01198

**Published:** 2017-07-18

**Authors:** Sandrine Zufferey, Pascal M. Gygax

**Affiliations:** ^1^Institute of French Language and Literature, University of Bern Bern, Switzerland; ^2^Department of Psychology, University of Fribourg Fribourg, Switzerland

**Keywords:** discourse connectives, discourse relations, language processing, language transfer, French as a foreign language

## Abstract

Discourse connectives are often reported to be difficult for second language learners, yet the causes of these difficulties are still not fully understood. In this paper, we test the ability of German-speaking learners to process and understand a connective with a complex form-function mapping in their L2-French, namely “en effet,” a connective that does not have an exact translation equivalent in their L1-German. We assess learners' competence both in an on-line processing experiment and an off-line judgment task. We argue that one of the interesting specificities of “en effet” is that the two coherence relations that it conveys cannot equally be conveyed implicitly. This case study therefore provides some information about advanced learners' sensitivity to the necessity of explicitly marking a coherence relation by the use of a connective. Our results indicate that advanced learners do not perceive the difference between relations that need and need not be marked by a discourse connective and have not acquired the complex form-function mapping of “en effet.” We argue that these difficulties cannot be attributed to negative transfer effects, but reflect general limitations in proficiency.

## Introduction

Discourse connectives are lexical items—like *because, if* and *when* in English—that explicitly indicate the coherence relation (i.e., causality, condition, etc.) linking discourse segments (Halliday and Hasan, 1976; Mann and Thompson, 1988; Sanders et al., 1992). Discourse connectives play a crucial role for language processing and comprehension for adult native speakers (e.g., Murray, 1997; Sanders and Noordman, 2000). Even young readers and poor readers do benefit, in terms of comprehension, from the presence of connectives for the online processing of discourse relations (Mouchon et al., 1995; Cain and Nash, 2011; van Silfhout et al., 2014). Thus, connectives play a crucial role for speakers' language competence.

In the field of second language learning, a recurrent observation in the literature is that connectives represent an area of difficulty, even for advanced learners. Many studies provide an analysis of elicited and natural written productions (mostly from advanced learners), and all of them report many cases of misuses as well as under- and over-uses of some connectives (Crewe, 1990; Field and Yip, 1992; Milton and Shuk-Ching Tsang, 1993; Lamiroy, 1994; Granger and Tyson, 1996; Altenberg and Tapper, 1998; Bolton et al., 2002; Müller, 2005; Tapper, 2005; Degand and Hadermann, 2009). These studies are, however, not sufficient to explain the causes of learners' difficulties, because they rely solely on production data. To our knowledge, very few studies have assessed learners' understanding of discourse connectives in controlled experiments. One study has demonstrated that advanced learners are able to detect non-native uses of connectives during online reading in L2-English, even when the misuse corresponds to licensed uses of similar connectives in their L1 (French and Dutch) (Zufferey et al., 2015). In an eye-tracking experiment, learners and native speakers did not differ in their reading patterns of sentences like (1) and (2) containing non-native uses of connectives typically produced by French- and Dutch-speaking English-learners (both non-native uses were identified on the basis of corpus data).

(1) The kids don't look tired today. When they don't sleep now, we can go out for a walk.
(2) People had a very different reaction to the president's speech. If in New-York most people agreed with him, in Texas people were appalled.

By contrast, the same learners fell prey to negative L1 transfer when performing a judgment task involving the same sentences. Dutch-speaking learners were specifically blind to the typical non-native use of the English connective *when* to convey a conditional instead of a temporal relation, and French-speaking learners were specifically unable to detect the non-native use of the English connective *if* to convey a contrastive instead of a conditional relation. Both groups of learners did, however, not differ compared to native speakers with the other non-native uses. Another study targeting off-line comprehension (i.e., not during reading but after reading a text) found that readers benefit from the presence of connectives, as their ability to answer questions about its content increases when connectives are used (Degand and Sanders, 2002). Taken together, these two comprehension studies indicate that advanced learners may have developed the intuitive ability to use connectives for text processing and comprehension, even though they still rely on L1 rules when producing explicit grammaticality judgments. However, these studies focused on two specific cases: the processing of connectives conveying a semantically incoherent relation, and the ability to use connectives' meaning to increase textual comprehension.

In this paper, we probe learners' mastery of connectives further by testing their ability to process and understand one connective that has a complex form-function mapping, namely the French connective “en effet” that either encodes a causal or a confirmation relation depending on context. We expect that German-speaking learners of French will experience difficulties with this connective, because its complex mapping with causal and confirmation relations is language specific. In German, these relations map to different connectives, namely “in der Tat” for confirmation relations and “denn” or “nämlich” for causal relations. There is preliminary evidence that the connective “en effet” is indeed particularly difficult for non-native speakers, as it is the most frequently looked-up French word on the Linguee^1^ online bilingual dictionaries.

Zufferey et al. (2015) demonstrated that learners experience difficulties in offline tasks when they are dealing with connectives in L2 that are the closest translation equivalent of connectives that have a complex form-function mapping in their L1. More specifically, learners have difficulties recognizing that one of the functions that they link to a connective in their L1 is not similarly encoded in their L2. For example, French-speaking learners of English erroneously associate the conditional and the contrastive meanings of the French connective “si” to the English connective “if,” even though “if” maps only with the conditional meaning of “si.” In this paper, we assess the hypothesis that learners also have difficulties when they have to encode several relations into one connective in L2, but these relations map with different connectives in their L1.

There is also another interesting dimension of learners' competence that can be tested through the case study of “en effet.” The two relations encoded in this connective have a very different potential for implicitness. In other words, while causal connectives are often optional and causal relations can easily be reconstructed by inference when the two segments are simply juxtaposed, confirmation relations must be marked explicitly by a connective much more often in order to be understood (Zufferey and Gygax, 2016). By comparing the way learners understand and process sentences with and without “en effet,” we will also get a glimpse of learners' sensitivity to the necessity to mark some discourse relations explicitly more often than others. So far, this question remains underexplored in the literature. It is also all the more relevant that from a cross-linguistic perspective, languages vary a lot in the explicit vs. implicit marking of discourse relations (e.g., Zufferey, 2016) and these differences could lead to negative transfer effects.

## The connective *en effet* and its translations in german

The connective “en effet” is a frequent lexical item in French. Studies focusing on this connective (Iordanskaja and Mel'cuk, 1999; Rossari, 2002; Charolles and Fagard, 2012; Danlos, 2012; Zufferey, 2016; Zufferey and Gygax, 2016) all indicate that *en effet* is ambiguous between a relation of causality as in (3) and a relation of confirmation as in (4).

(3) Sarah est heureuse. En effet, elle a réussi ses examens.
  Sarah is happy. CONNECTIVE she passed her exams.
(4) Les parents de Sarah pensaient qu'elle réussirait ses examens. Et en effet elle les a réussis.
  Sarah's parents thought that she would pass her exams. And CONNECTIVE she did pass them.

When *en effet* conveys a causal relation, it is always used in clause initial position. It can however also be used in clause medial or clause final positions when it conveys a confirmation relation. Several studies (Charolles and Fagard, 2012; Danlos, 2012) note that when *en effet* is used to convey a confirmation relation in clause-initial position, it is preceded by the connective “et” (the French equivalent of the English *and*). The addition of “et” before the connective pragmatically indicates a temporal sequence that is not compatible with a causal relation, in which the cause following the connective typically occurs before the consequence presented in the first segment (Charolles and Fagard, 2012). Therefore, the locution *et en effet* is an effective way to ensure that a confirmation rather than a causal relation is conveyed in clause initial position.

An important difference between these two functions of “en effet” is that these relations are not equally easy to infer when they are left implicit in a text. On the one hand, causal relations are easily understood when they are conveyed implicitly (Murray, 1997; Sanders, 2005) and they are indeed often left implicit in corpus data (Asr and Demberg, 2012). On the other hand, confirmation relations are not easily reconstructed when they are left implicit, because they involve a perspective shift between the narrative perspective of an external speaker in the first segment and the speaker's own perspective in the second segment. In the case of (4), the external perspective is that of Sarah's parents in the first segment, while the speaker's own perspective is presented in the second segment. This discrepancy has been confirmed in a processing experiment involving native speakers, who encountered a processing delay at the end of implicit relations of confirmation (4) but not while reading causal relations (3) (Zufferey and Gygax, 2016).

This difference has also been found to affect the translations of “en effet” in several target languages like English, German, and Spanish, as confirmation relations are translated explicitly significantly more often in all of them, independently of the range of translation equivalents provided by the target language system (Zufferey, 2016; Zufferey and Gygax, 2016). Of particular interest for this paper is the case when French is translated into German. In Table 1, we report the number of occurrences of each translation equivalent per discourse relation for the 500 occurrences of “en effet” that were randomly extracted from the Europarl corpus (from Zufferey and Gygax, 2016).

**Table 1:** Annotation and translation spotting of German translations.

	**zero**	***denn***	***nämlich***	***in der Tat***	**other**	**Total**
Cause	153 (31%)	75 (15%)	57 (11%)	32 (6%)	103 (21%)	420 (84%)
Confirmation	10 (2%)	6 (1%)	4 (1%)	28 (6%)	32 (6%)	80 (16%)
Total	163 (33%)	81 (16%)	61 (12%)	60 (12%)	135 (27%)	500 (100%)

*Denn roughly corresponds to because, nämlich to in fact, and in der Tat to as a matter of fact (our en effet)*.

Table 1 illustrates perfectly the idea that implicit translations are more often associated with causal then with confirmation relations. The percentage of implicit translations is 31% when *en effet* conveys a causal relation (153 out of 500 occurrences) and only 2% when it conveys a confirmation relation (10 out of 500 occurrences). In addition, Table 1 indicates that “zero” is by far the most frequent translation equivalent of *en effet* when it conveys causal relations, compared to each of the other translation choices. In other words, for German-speakers, a natural way to convey a causal relation in contexts where *en effet* is used in French is to leave the relation implicit.

Arguably, the subjective causal connective *denn* is not more frequent as a translation equivalent for causal relations conveyed by *en effet* because the specificity of *en effet*—as a causal connective—is to occur in sentence initial position, with separate sentences for the two related segments. The separation of segments into two different sentences makes juxtaposition (i.e., two successive sentences linked only by a full stop) a more attractive translation choice in this syntactic context, compared to causal connectives that are typically used in sentence-medial position such as *car* and *parce que*. The subjective causal connective *denn* in German is therefore more closely associated with the subjective causal connective *car* in French (Pit, 2007). By contrast, when *en effet* conveys a confirmation relation, its most frequent translation equivalent is the similar locution *in der Tat* in German. In fact, German-speaking learners can use this close equivalence and benefit from positive transfer in order to process and understand the meaning of *en effet* in confirmation relations.

## Processing of *en effet* by L1 and L2 readers: hypotheses

In an on-line reading experiment with native speakers, Zufferey and Gygax (2016) found two effects related to the reading of explicit and implicit relations of cause and confirmation conveyed by *en effet*. First, readers were faster to process sentences in the presence of an explicit connective than in its absence (i.e., implicit relations), independently of the relation conveyed by *en effet*. This effect was already visible at the level of the words immediately following the connective. The second effect was a slower processing time for implicit confirmation relations compared to explicit ones. This effect was most visible at the end of the sentence, when readers try to integrate the semantic content of the two segments into a coherent relation.

Contrary to native speakers, German-speaking learners could fail to process sentence stems introduced by an explicit connective faster and also fail to notice the loss of coherence produced by implicit confirmation relations. First, in their L1-German, causal relations introduced by the sentence initial connective in French are more often left implicit rather than conveyed explicitly by inserting a clause medial connective such as *denn*. In other words, German-speaking L2-French learners may not benefit from the presence of explicit connectives in relations that are implicit in their L1-German due to negative transfer. Second, the integration of words that encode procedural meaning—such as connectives and pronouns—during online reading have been shown to be difficult even for advanced learners, and even independently of transfer effects. Roberts et al. (2008) reported that German-speaking learners perform on a par with native Dutch speakers on the resolution of ambiguous subject pronouns in an off-line task, something that they attribute to the similarity between Dutch and German, that are both non-null subject languages. A similar positive transfer effect was however not found in an eye-tracking experiment, as German-speaking learners showed an online processing disadvantage compared to native speakers. The authors concluded that this effect was due to processing limitations in L2 that prevented positive transfer from taking place. We argue that similar processing limitations might also prevent learners from detecting the loss of coherence in implicit confirmation relations, even though these relations are mostly marked explicitly in German. As a consequence, the confirmation relation effect found in Zufferey and Gygax (2016) might be absent in L2-French learners due to the cognitive overload observed during online processing. We directly tested these hypotheses using both on-line (reading times) and off-line (acceptability rating) measures on L1-French and L2-French speakers. In a nutshell, in the online reading experiment, we expect L2-French speakers not to detect the loss of coherence for implicit confirmation relations and to process similarly causal and confirmation relations. In the offline experiment, we expect L2-French speakers not to rate explicit relations as more coherent than implicit relations.

## Experiment 1: online processing of implicit and explicit causal and confirmation relations conveyed by *en effet*

### Methods

#### Participants

Participants were 31 native French speakers (L1-French) and 29 advanced German-speaking learners of French (L2-French), all students from the University of Fribourg in Switzerland (mean age: 23, range 18–42, 45 female). The group of German-speaking learners reached an average C-test score (Coleman, 1994) in French of 83.33% (*SD* = 10.69%), testifying of their advanced learners' level in French^2^, native speakers' scores on the C-test habitually ranging—depending on the situation and context—from 80 to 96% (e.g., Jafarpur, 1995; Huhta, 1996). On this particular test, MA students scored at 85% (Coleman, 1994) a very similar score to that of our participants.

The experiment was approved by the University's ethics committee, and all participants had granted their written informed consent.

#### Materials

The material for this experiment is the same as is the one used by Zufferey and Gygax (2016) to test native speakers of French. All participants read 40 test items, created in four different versions. For all items, the critical segment was the same, but two different pre-critical sentences were inserted in order to create either a relation of confirmation (5) or causality (6).

(5) Susanne avait l'impression qu'il lui manquait quelque chose.
  Susanne felt that she had lost something.
  Et en effet, elle a oublié son portefeuille dans le bus.
  And CONNECTIVE she forgot her purse in the bus.
(6) Susanne ne fait manifestement pas attention à ses affaires.
  Suzanne is obviously rather careless with her belongings.
  En effet, elle a oublié son portefeuille dans le bus.
  CONNECTIVE she forgot her purse in the bus.

For both relations, one version of the experimental item contained the connective *en effet*, while another version contained an implicit relation as in (7) and (8).

(7) Susanne avait l'impression qu'il lui manquait quelque chose.
  Susanne felt that she had lost something.
  Elle a oublié son portefeuille dans le bus.
  She forgot her purse in the bus.
(8) Susanne ne fait manifestement pas attention à ses affaires.
  Suzanne is obviously rather careless with her belongings.
  Elle a oublié son portefeuille dans le bus.
  She forgot her purse in the bus.

In confirmation relations, the perspective shift between beliefs held by an external source and the speaker's own confirmation was systematized across all items by the insertion of a lexical marker explicitly indicating the source of belief in the pre-critical sentence. In example (5) the indication is “*Suzanne felt*.” In the case of causal relations, one of the difficulties of having an implicit relation is that readers may interpret them as objective forward cause-consequence relations instead of subjective backward consequence-cause relations. For example, in (6) the fact that Suzanne is careless could be interpreted as a cause and her forgetting her purse as a consequence. In order to prevent readers from inferring a forward cause-consequence relation, a lexical marker of subjectivity was systematically included in all pre-critical sentences (as shown in Pander-Maat and Degand, 2001; Degand and Pander Maat, 2003). For example, in (6), this marker is the epistemic adverb *obviously*. This marker leads the reader to interpret the first segment as a subjective conclusion rather than an objective cause.

The critical clause was divided into three reading segments, designed as follows. The first segment contained the subject and verb of the clause, and was on average made of 3 words (*SD* = 0.7), corresponding to 12 characters (*SD* = 4). The second segment contained the complement (direct object) of the first clause, and was on average made of 3 words (*SD* = 0.8) corresponding to 15 characters (*SD* = 4.2). The last reading segment contained a syntactically optional adjunct that was on average made of 2.7 words (*SD* = 0.7), corresponding to 12 characters (*SD* = 4). A list of all experimental items is provided in Appendix 1 (Supplementary Material).

#### Procedure

The experiment was run using the ZEP self-paced reading software (Veenker, 2013). The participants were tested individually and each session began with written instructions about the experiment, followed by a training phase, in which participants read sentences similar to the experimental and filler items. At the end of the training phase, they were given the opportunity to ask questions to the experimenter before the actual experiment began. All trials began with a fixation point indicating where the sentence would start to appear. Participants could progressively read the sentences—segment after segment—by pressing the space bar. The sentences were divided into seven reading segments, appearing consecutively on a computer screen, as illustrated in (9).

(9) [Suzanne avait 1] [l'impression qu'il lui 2] [manquait quelque chose. 3] [Et en effet, 4] [elle a oublié 5] [son porte-monnaie 6] [dans le bus. 7]
  [Suzanne felt 1] [that she had 2] [lost something. 3] [CONNECTIVE, 4] [she forgot 5] [her purse 6] [in the bus. 7]

The previous segments of the sentence disappeared from the screen as the readers went on to the next one. This design was meant to prevent participants from displaying the whole sentence by pressing several times on the space bar before starting to read it.

The stimuli were divided into four lists using a Latin square design, with only one version of a particular dialogue included per list. The order of presentation was randomized. In addition, 32 filler items containing object and subject relative clauses were inserted in each list. Verification statements were inserted randomly after 50% of the trials, in order to assess participants' level of attention. For example, the (true) statement following (9) was: “Suzanne a oublié son porte-monnaie” [*Suzanne forgot her purse*]. When such statements occurred, participants were asked to click on a “true” or “false” button appearing below the statements to enter their answer. Items that triggered an incorrect response from the participant were removed from the analyses. Such incorrect answers represented 2.84% of the data. No time constraint was imposed for the task, and participants completed it, on average, in about 15 min.

### Results

Only the reading times for the second segment were compared across all conditions, because the first sentence varied across the two types of relations (confirmation vs. cause). These regions correspond to the reading segments 5–7, as indicated in (9) and repeated in (10) for convenience.

(10) [She forgot 5] [her purse 6] [in the bus 7].

In all analyses, reading times that were three standard deviations above or below each participant's means were replaced by their cut-off values. They represented 1.97% of the data (1.88% for Segment 5; 1.92% of Segment 6 and 1.92 of Segment 7).

Since we had clear hypotheses as to the different three target segments (i.e., Segments 5, 6, and 7) in the on-line data, we present three separate analyses. Mean reading times per critical segment and per condition are reported in Table 2.

**Table 2:** Mean reading times and standard deviations (in brackets) per condition and per segment in milliseconds.

**Segment reading times**					
**Group**	**Relation**	**Connective**	**Segment 5**	**Segment 6**	**Segment 7**
			**Subject and verb**	**Complement**	**Final adjunct**
*L1-French*	*Cause*	Explicit	764	815	844
			(479)	(495)	(604)
		Implicit	808	811	863
			(517)	(556)	(613)
	*Confirmation*	Explicit	744	836	832
			(435)	(573)	(558)
		Implicit	789	792	918
			(493)	(468)	(596)
*L2-French*	*Cause*	Explicit	1,056	1,191	1,130
			(524)	(626)	(615)
		Implicit	1,076	1,122	1,164
			(535)	(582)	(676)
	*Confirmation*	Explicit	964	1,188	1,162
			(465)	(541)	(606)
		Implicit	1,069	1,102	1,105
			(587)	(498)	(553)

In order to include both participants and items as random factors in all analyses, therefore avoiding the “language-as-fixed-effect-fallacy” by separating F1 and F2 analyses (Clark, 1973; see Brysbaert, 2007 for an initial presentation of the controversies related to separate F1 and F2 analyses), data were analyzed by fitting linear mixed-effects models using the R software (R Development Core Team, 2010, version 3.1.2). Linear mixed-effects models are particularly useful, not only as they enable us to avoid the “language-as-fixed-effect-fallacy,” but they also allow us to perform analyses that account for missing values. In a nutshell, the first step in linear mixed-effects analyses is to compare models that have different fixed and random effects. The comparison generates a maximum likelihood ratio, which tells us which model best fits our data (using a χ^2^ distribution). The maximum likelihood model is then analyzed to document main and interaction effects of the fixed factors, in a similar way as a traditional ANOVA would do. Note that degrees of freedom need to be adjusted (e.g., with Kenward–Roger approximation) to control for Type I errors. In this present paper, models were tested using the *lmer()* function of the *lmer4* package of R, and model comparisons were assessed using the *anova()* function, which calculate the Chi-square value of the log-likelihood in order to evaluate the difference between models, following Baayen's (2008) procedure. Finally, the *p*-values, *F*-values, and degrees of freedom estimates were obtained with the *mixed()* function (from the *afex* package by Singmann et al., 2015)^3^.

In this experiment, we were particularly interested in the interaction effects of Connective (Explicit vs. Implicit) and Relation (Confirmation vs. Cause), as in Zufferey and Gygax (2016), but most importantly in association with Group (L1-French or L2-French). Therefore, we initially compared a model that only encompassed items and participants as random factors (i.e., our random model), to that a maximal model encompassing Connective, Relation, and Group as fixed factors (main and interaction effects), Relation as random slope and intercept for items, and Connective as random slope and intercept for participants^4^. If the model was improved, we then removed any factor that was not significant, and further tested if the model was improved. If it did not, we considered the maximal model as the final one (see Appendix 2 in Supplementary Material for a summary of the final models of each segment).

#### Segment 5

Adding Connective, Relation, and Group to the initial model, Relation as random slope and intercept for items, and Connective as random slope and intercept for participants, significantly improved our random model, which only including items and participants as random factors, Δχ^2^ = 42.342, Δdf = 11, *p* < 0.001. Removing any of the factors did not improve the model. We therefore kept the model including all factors as our final model.

The final model, including Group, Connective, and Relation as fixed factors, items, and participants as random intercepts and Connective as random slope by participant, showed three main effects. First, there was an obvious effect of Group, *F*_(1, 56.96)_ = 11.36, *p* < 0.001, showing that participants were faster in L1-French (*M* = 776; *SD* = 482) than in L2-French (*M* = 1041; *SD* = 530). Second, there was a main effect of Connective, *F*_(1, 55.97)_ = 8.57, *p* < 0.01, showing that when the segment was introduced by an explicit connective, participants were faster to read it (*M* = 885 ms; *SD* = 495) than when no connective was present (*M* = 936 ms; *SD* = 551). Third and finally, there was an effect of Relation, *F*_(1, 37.43)_ = 4.00, *p* < 0.05, showing that when the segment was characterized by a confirmation relation, participants were faster to read it (*M* = 893 ms; *SD* = 514) than when it was a causal relation (*M* = 927 ms; *SD* = 532). There was no interaction effect.

#### Segment 6

Adding Connective, Relation, and Group to the initial model, and Connective as the random slope by participant, significantly improved our random model, which only included items and participants as random factors, Δχ^2^ = 28.556, Δdf = 11, *p* < 0.01. As Relation did not appear to show any main nor interaction effect, we removed it and compared the resulting model to the maximal one. It did not, however, improve the model, Δχ^2^ = 2.828, Δdf = 6, *ns*. We therefore considered the maximal model as the final one.

The final model, including Group, Connective, and Relation as fixed factors, Relation as random slope and intercept for items, and Connective as random slope and intercept for participants, showed two main significant effect. First, there was an effect of Group, *F*_(1, 57)_ = 16.35, *p* < 0.001, showing that participants were faster in L1-French (*M* = 813; *SD* = 524) than in L2-French (*M* = 1151; *SD* = 564). Second, there was an effect of Connective, *F*_(1, 56.34)_ = 7.34, *p* < 0.01, showing that, somehow surprisingly, participants were faster to read segment 6 when the connective was absent (*M* = 957; *SD* = 550) than when present (*M* = 1011; *SD* = 589). There was no other main or interaction effect.

#### Segment 7

As for the segments 5 and 6, the initial random model improved when all three fixed factors and the random intercepts and slope were added, Δχ^2^ = 23.688, Δdf = 11, *p* < 0.05. As all factors showed some significant effects, we considered the maximal model as the final one.

**Figure 1:**
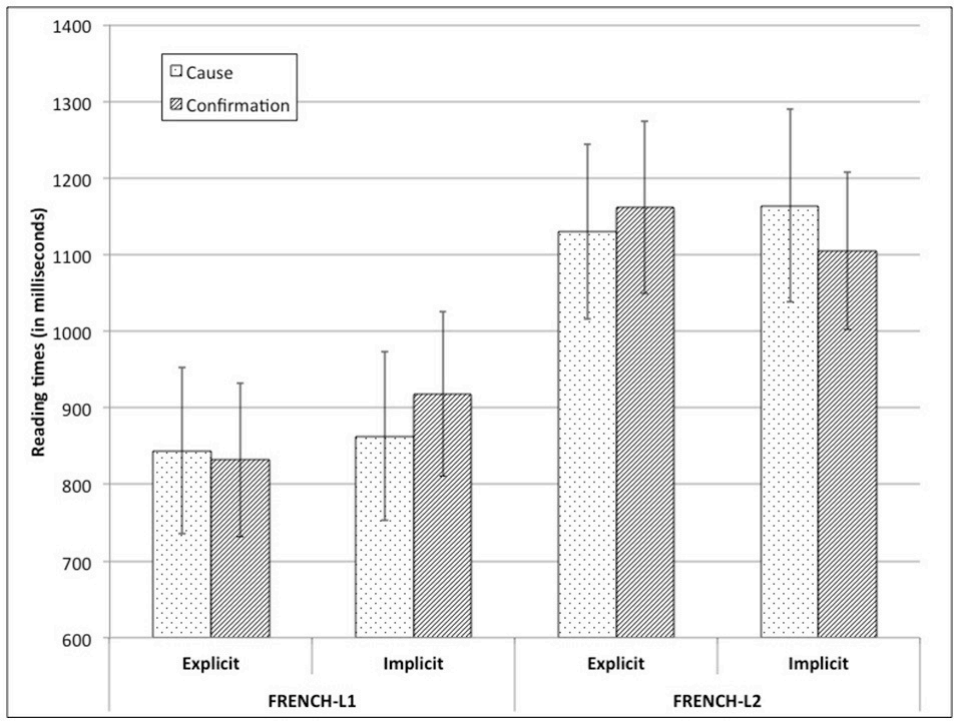
Mean reading times and standard errors of Segment 7 (in ms).

The final model, including Group, Connective, and Relation as fixed factors, Relation as random slope and intercept for items, and Connective as random slope and intercept for participants, showed two interesting effects. First, there was an obvious effect of Group, *F*_(1, 56.98)_ = 8.25, *p* < 0.01, showing that participants were faster in L1-French (*M* = 864; *SD* = 593) than in L2-French (*M* = 1140; *SD* = 614). Second, there was an important three-way interaction effect, *F*_(1, 2112.58)_ = 4.32, *p* < 0.05, as illustrated in Figure 1.

To further explore this interaction, we analyzed the data separating language groups. When considering L1-French speakers, the only significant effect was that of Connective, the segment being read faster when the relation was explicit than when it was implicit, *F*_(1, 27.55)_ = 4.45, *p* < 0.05. When analyzing L2-French speakers, the main effect of Connective was not significant, *F*_(1, 27.83)_ < 1, *ns*, yet there was a close-to-significant Connective by Relation interaction effect, *F*_(1, 1037.64)_ = 2.72, *p* = 0.10. Although, pair-wise comparisons showed no significant differences, when the relation was explicit, the segment was read numerically faster than when the relation was of confirmation. This was the opposite for implicit relations (see Figure 1).

### Discussion

As in Zufferey and Gygax (2016), readers were faster to read a segment (i.e., segment 5) introduced by an explicit connective than when the relation was implicit. However, this was the case for all our participants, independently of their language proficiency. Learners of French did not seem to suffer from negative L1-German transfer, as the explicit marking with a connective generated a faster processing time than when the connective was absent. For all readers, an explicit connective seemed to ease the mental processing of related segments, thus indicating that for these relations, the connective provides an immediately useful cue. This pattern is similar to the one observed in Zufferey and Gygax (2016).

Somehow surprisingly, and in contrast to Segment 5, participants read Segment 6 more slowly when introduced by a connective than when the relation was implicit. This effect could be accounted for by different processes. A tentative explanation would be a trade-off effect from reading speed of Segment 5. In other words, readers slowed-down while processing Segment 6, as a consequence of parsing Segment 5 more rapidly. Note that although this effect was not significant in Zufferey and Gygax (2016), it was numerically similar. This effect could also signal some encoding processing. Some authors (e.g., Gillioz and Gygax, 2017) have in fact argued that in some cases, slower reading times signal deeper encoding processes. In a series of experiments on emotion inferences, Gillioz and Gygax (2017), for example, showed that participants took longer to read textual elements that were most salient in the context of the experimental narratives. When asked to recall these textual elements after the experiment, the authors found that the slower these elements were read in the reading experiment, the more likely they were to be recalled. They interpreted these results as the signal of a possible encoding mechanism, needing more cognitive resources (i.e., as do *deeper* cognitive processes), hence generating slower reading times. Still, most interesting were the effects of Segment 7, inasmuch as they were involving language groups.

In Segment 7, native L1-French were slower to read confirmation relations without connective than with it, as predicted by the perspective shift hypothesis. Accordingly, perspective shifts introduce a form of discontinuity in the text that has to be marked explicitly. This effect seemed to be driven by the language group, the difference in reading times between explicit and implicit confirmation relations being much smaller for the L2-French group. This difference between L2-French learners and native speakers cannot be due to negative L1 transfer, as these relations are also mostly marked by explicit connectives in German translations of French sentences containing confirmative uses of “en effet.”

Learners' smaller difference in reading times between explicit and implicit relations of confirmation may indicate that they did not seem to notice the loss of coherence coming from the absence of connectives the way native speakers did. This could be due to a performance limitation caused by the burden of on-line reading, especially in a self-paced reading context that places greater demands on working memory, due to the impossibility to go back to previous segments and reread them.

For this reason, in Experiment 2, we probed further learners' comprehension of the two discourse relations conveyed by “en effet” in an off-line coherence judgment task. In a study comparing learners' on-line and off-line ability to handle the meaning of connectives (Zufferey et al., 2015), a major difference was found between an on-line reading task and an off-line judgment task in learners' ability to spot non-native uses of connectives. A similar discrepancy between on-line and off-line tasks was also reported by Roberts et al. (2008) in the case of overt pronouns. In order to disentangle the role of processing limitations from proficiency limitations, we also included an off-line judgment task after the reading experiment. If learners do not display an effect at the end of the sentence during reading but still evaluate explicit confirmation sentences as more coherent than implicit one, we could conclude that processing limitations caused the on-line group difference. If on the other hand learners also fail to evaluate explicit confirmation relations as more coherent than implicit ones, this would be indicative of a lack of integration of the procedural meaning of connectives in L2.

## Experiment 2: coherence judgment task

### Methods

#### Participants

The participants were the same as in Experiment 1.

#### Materials

The experimental sentences chosen for this post-Experiment 1 task were a random selection of five experimental items per condition: explicit vs. implicit and confirmation vs. cause, leading to a total of 20 sentences. Each participant thus saw 5 sentences per condition. The items were randomized and inserted in four different lists. If an item was presented in a condition in a list, that item was part of another condition one of the other lists. Participants were randomly assigned to one list.

#### Procedure

After completing Experiment 1, participants were told that they would see again a selection from the sentences that they had just read. This time, their task was to assess the coherence of the relation between the two sentences of each item on a five point Likert scale: 1 = “very incoherent,” 2 = “rather incoherent,” 3 = “neutral,” 4 = “rather coherent,” 5 = “very coherent.” No time constraint was imposed for this task, and participants completed it in 5–10 min.

### Results

The models were analyzed considering participants' judgment on the five-point scale described above. As for the online measures, we first considered an initial random model, and then compared it to the maximum model (with all fixed factors, random intercepts, and a random slopes). We then removed any factor that was not significant, and further tested if the model was improved. If it did not, we considered the maximal model as the final one. Results are reported in Table 3.

**Table 3:** Mean coherence judgment scores (standard deviations in brackets).

**Judgment**			
**Group**	**Relation**	**Connective**	**Relation between sentences**
*L1-French*	*Cause*	Explicit	4.22
			(0.99)
		Implicit	4.03
			(1.11)
	*Confirmation*	Explicit	3.74
			(1.26)
		Implicit	3.46
			(1.15)
*L2-French*	*Cause*	Explicit	3.74
			(1.32)
		Implicit	4.05
			(1.15)
	*Confirmation*	Explicit	3.74
			(1.44)
		Implicit	4.01
			(1.16)

As for all segments in the on-line measure analysis, the initial random model improved when all three factors were added, along with their random slopes and intercepts, Δχ^2^ = 60.218, Δdf = 11, *p* < 0.05. As all factors showed some significant effects, we considered the maximal model as the final one.

The analysis of the final model showed two significant effects. First, there was a Relation by Group interaction effect, *F*_(1, 1026.66)_ = 14.23, *p* < 0.001. For the L1-French group, participants did provide a higher coherence score when causal relations were presented (*M* = 4.13; *SD* = 1.05) than when confirmation relations were presented (*M* = 3.60; *SD* = 1.21, *p* < 0.001). For the L2-French group, however, this was not the case (confirmation: *M* = 3.87; *SD* = 1.27; cause: *M* = 3.90; *SD* = 1.25; *ns*). Finally, there was also a Connective by Group interaction effect, *F*_(1, 55.96)_ = 15.90, *p* < 0.001, which was qualified by a significant difference for L2-French learners, who judged sentences with explicit connectives as less coherent (*M* = 3.74; *SD* = 1.38) than those with no connectives (*M* = 4.03; *SD* = 1.15; *p* < 0.001). As for L1-French, the effect was reversed: L1-French participants judged sentences with explicit connectives as more coherent (explicit: *M* = 3.98; *SD* = 1.16) than those with no connectives (*M* = 3.75; *SD* = 1.16; *p* = 0.08).

### Discussion

Quite logically, independently of their explicit or implicit status, L1-French speakers provided generally higher ratings for causal relations than confirmation ones. This result can be attributed to the frequency of the uses of “en effet” in naturally occurring data. In the corpus data reported by Zufferey and Gygax (2016), over 80% of the uses of “en effet” were causal. But this effect was not found for learners, who seemed to be rather impervious to the type of relation conveyed by “en effet,” as they provided identical coherence scores to explicit causal and confirmation relations.

The lack of difference in judgments given by L1-French speakers between explicit and implicit relations conveyed by “en effet” in confirmation relations underline the necessity to perform both on-line and off-line tasks to assess the comprehension of lexical items such as connectives. Indeed, because connectives encode procedural rather than conceptual meaning (Sperber and Wilson, 1993; Blakemore, 2002) unlike most other lexical items, their meaning is notoriously difficult to bring to consciousness even for native speakers (Wilson, 2011). Their intuitions about the felicitous and infelicitous uses of connectives is therefore much more visible in tasks targeting on-line reading. In these tasks, readers have repeatedly been found to react to inappropriate uses of connectives (Traxler et al., 1997; Canestrelli et al., 2013; Zufferey et al., 2015).

L2-French learners' judgment perfectly matched the picture provided by their on-line reading data. Indeed, learners' lesser reaction (in terms of slower reading times) to the loss of coherence provoked by implicit confirmation relations was reflected in their preference for implicit over explicit uses of connectives in the coherence judgment data. These results, taken together with the reading times, indicate that L2-French learners really struggle with the connective *en effet*. Most importantly, L2-French learners consider *any* sentence with a connective as less coherent than without, independently of the relation carried by the connective.

In sum, results from this off-line judgment task were in line with the patterns observed in the on-line reading experiment (with some interesting differences) and confirmed that learners do not take into account the information provided by connectives in L2 as they systematically prefer implicit over explicit relations, even when a similar marking device exists in their L1. It seems therefore that learners have not fully acquired either uses of *en effet*, as they had no preference for the most frequent causal uses similar to the one observed for native speakers.

## General discussion

In this paper, we tested advanced learners' processing of one French connective with a complex form-function mapping, namely “en effet.” We assessed their reading and comprehension of confirmation and causal relations, both when they were conveyed explicitly with this connective or implicitly. We argued that the two relations that can be conveyed by “en effet” are not equal in terms of their capacity to be conveyed implicitly. While causal relations can be made implicit without a loss of coherence, confirmation relations must be marked explicitly, because they involve a perspective shift that breaks textual continuity (Zufferey and Gygax, 2016).

Results obtained with native French speakers confirmed these predictions. In the on-line reading experiment (i.e., Experiment 1), they displayed longer reading times at the end of the sentence when confirmation relations were implicit compared to explicit ones. The same pattern was not found, however, for causal relations, reflecting their higher potential for implicitness. Similarly, in the off-line judgment task, native speakers judged explicit cases of confirmation relations as more coherent than implicit ones. They did also judge causal relations as globally more coherent than confirmation ones. As mentioned earlier, this effect can easily be attributed to the markedly higher frequency of these relations for the connective “en effet” in corpus data (86% for causal relations vs. only 14% of confirmation relations, see Table 1).

Across both experiments, learners proved not to be as sensitive as native speakers to the loss of coherence related to the implicit communication of confirmation relations and did not seem to make a difference between causal and confirmative uses of *en effet*. Indeed, L2-French learners did not react to the lack of connective in confirmation relations as L1-French natives did in the on-line experiment. In the off-line judgment task (Experiment 2), L2-French learners even rated implicit relations (both causal and confirmation) as more coherent than explicit ones (which was the reverse for L1-French speakers). These results suggest that L1-German learners of French are not sensitive to the necessity to explicitly mark confirmation relations in order to avoid a loss of coherence in their L2-French and do not master the complex form-function mapping of *en effet*.

These results thus raise the question of what type of meaning learners have encoded for the connective *en effet*. On the one hand, one could argue that learners have only acquired its causal meaning because it is a lot more frequent in the input, but fail to integrate its confirmative meaning. On the other hand, one could argue that learners have integrated the confirmative meaning of *en effet* as a result of positive transfer from the closely related connective *in der Tat* in German. This is not the case for its causal meaning, because it does not have a clear one-to-one mapping with German causal connectives. The fact that L2-French learners did not rate explicit causal and confirmation relations differently in Experiment 2 contradicts both these hypotheses. Indeed, if learners understood *en effet* to be a causal connective, they should have rated explicit confirmation relations as less coherent than explicit causal relations, because a causal meaning could not be inferred in this context. This was not the case in our data. Similarly, learners did not demonstrate a preference for explicit confirmative uses over causal uses of *en effet*. Our results thus imply that learners do not seem to master any of two uses of *en effet*. This finding raises interesting issues for the way learners integrate the meaning of words that encode procedural meaning in a second language. Indeed, *en effet* is a very frequent lexical item in French and advanced learners must have come across it frequently, but still do not seem to understand its meanings. This conclusion is corroborated by the observation that *en effet* is the most frequently looked up French word in Linguee bilingual dictionaries, indicating that non-native speakers do indeed not know how to use it. In future work, similar experiments should be conducted with other connectives that possess a complex form-function mapping, in order to determine the extent of this problem for learners. Several studies focusing on corpus data did underline that learners seem to avoid some connectives, without providing explanations for this phenomenon (e.g., Granger and Tyson, 1996). Future experimental studies should seek to determine whether complex form-function mappings cause greater difficulties for learners^5^.

Our experiments were designed to assess the role of negative transfer vs. general limitations in proficiency as factors explaining learners' difficulties with connectives. Based on our results, we conclude that limitations in proficiency rather than negative transfer seems to cause learners' lack of sensitivity to the uses of *en effet*. Indeed, in the German translations of “en effet” found in the 500 occurrences extracted from the Europarl corpus, confirmation relations were very predominantly translated explicitly in German, by the use of connectives such as the close translation equivalent “in der Tat.” Even though German also possesses a subjective causal connective “denn” that matches the causal uses of “en effet,” many occurrences were left implicit in German. In fact, zero equivalent was by far the most frequent translation choice for causal uses of “en effet.” Thus, if learners failed to integrate the processing instructions conveyed by “en effet” due to negative L1 transfer, these problems should be found for causal uses of this connective. The only element in our data suggesting such negative influence is learners' lack of higher coherence rating for causal over confirmative uses of “en effet” in the judgment task. In our self-paced reading data, however, learners and native speakers were similarly influenced by the use of “en effet.” Both L1-French and L2-French speakers were faster to read sentences with connectives than without in the segment immediately following the connective. Thus, transfer does not appear to be the key factor explaining the divergences between native speakers and L2-learners.

The main differences between learners and native-speakers were found in their reading of confirmation relations (Segment 7) and in the judgments of relations with or without connectives. One could argue that the lack of coherence created by implicit confirmation relations is specific to French. As a result, learners' lack of sensitivity to the absence of connectives—or for that matter the lower coherence ratings of sentences with connectives—might mainly reflects a lack of competence with connectives in L2. In the reading experiment, this lack of sensitivity could be attributed to the high cognitive demands of on-line reading. However, in the off-line task, learners' consistent lack of sensitivity to the loss of coherence in implicit confirmation relations rules out this interpretation. Overall, learners marked as less coherent explicit discourse relations with the connective “en effet” independently of the relation conveyed by this connective, thus demonstrating their lack of sensitivity to the loss of coherence produced by implicit confirmation relations.

Future experiments should determine whether learners also lack sensitivity to the difference of coherence between explicit and implicit relations in L2 with connectives that have clear translation equivalents in their L1. For example, one could assess whether learners have a greater sensitivity to the loss of coherence produced by other relations such as concessive relations.

Our experiments also contributed to deepen our understanding of the way learners construct and understand discourse structure. Our results indicate that even at advanced stages of language learning, discourse structuring remains an area of difficulty for learners^3^. This conclusion is concordant with studies that have demonstrated advanced learners' lack of sensitivity to information structure in discourse (e.g., Park, 2011). In a nutshell, given the importance of mastering connectives in order to produce and understand discourse coherence, future experiments should seek to probe learners' sensitivity to the necessity to explicitly mark discourse structure across a wider range of discourse relations and connectives, and by comparing a wider range of language combinations.

## Ethics statement

The study was approved by the Ethics committee of the Department of Psychology from the University of Fribourg. Written consent was given by all participants.

## Author contributions

SZ and PG designed the experiment. SZ ran the experiment and wrote the first draft. PG wrote the method and result sections.

### Conflict of interest statement

The authors declare that the research was conducted in the absence of any commercial or financial relationships that could be construed as a potential conflict of interest.

## Footnotes

^1^http://www.linguee.fr/french-english/topfrench/1-200.html (page last consulted on June 1, 2017).

^2^Essentially, the *C*-test was used to ensure that our L2-French participants were sufficiently proficient to understand the materials presented to them. The *C*-test, which is particularly useful in its ease of implementation, has been extensively researched and has been shown to be a highly reliable objective measure of language proficiency (Eckes and Grotjahn, 2006). Importantly, *C*-tests have been shown to be highly correlated with standardized tests (e.g., the five competencies of the Test de Connaissance du Français, mirroring CEFR measures, Reichert et al., 2014). Note that we also removed from the analyses those items that participants did not respond to appropriately (i.e., 2.84% of the data) to avoid confounds related to a general lack of proficiency.

^3^*Post-hoc* comparisons were calculated with the *glht* function, with Tukey pair-wise comparisons with the Bonferonni correction.

^4^There is a relatively complex debate as to the choice of factors for defining the random effect structure in mixed linear models (e.g., Barr et al., 2013; Bates et al., 2015). On the one hand, overly simple random effects structures tend to produce too liberal tests for fixed effects, whereas over-specifications of random effects tend to be difficult to compute (e.g., convergence issues) and interpret (e.g., parameters' estimates). In our data, we initially tried to implement the maximal level of random structure, yet when the model did not converge, we removed, step-by-step, the random effects factors (and their interactions). The resulting models are those that converged and that made theoretical sense. Since implicit relations do activate relatively complex inference processes, a reasonable assumption to make is that, irrelevant of our fixed factor (i.e., Group), the effect of Connective (implicit vs. explicit) may well vary across participants (i.e., random slope and intercept). Likewise, since we used the same critical sentences to imply both causality and confirmation—pre-critical sentences varied in association to Relation—, it is reasonable to assume that some critical sentences might well show differences in their propensity to convey both meanings.

^5^Note that we have no indication as to the way connectives were taught to our L2-French learners. This might be a valuable piece of information in future studies.
