# Supplementary material for: Processing Connectives with a Complex Form-Function Mapping in L2: The Case of French “En Effet”
Source: Front Psychol. 2017 Jul 18;8:1198. doi: 10.3389/fpsyg.2017.01198 (PMC5514365; doi:10.3389/fpsyg.2017.01198)
Supplement: Supplementary file 1 [file Appendix1.pdf]

Appendix 1: List of experimental items

1.
Max pensait que sa femme avait une liaison.
Et en effet/Ø Elle a un amant depuis plusieurs mois.

Max ne doit pas être très proche de sa femme.
En effet/Ø Elle a un amant depuis plusieurs mois.

2.
Pierre pensait que le gardien était un excellent joueur.
Et en effet/Ø L’adversaire n’a pas réussi à marquer de but de toute la partie.

Le gardien de but est de toute évidence un excellent joueur.
En effet/Ø, l’adversaire n’a pas réussi à marquer de but de toute la partie.

3.
Emilie avait l’impression d’avoir froid au dos ce matin.
Et en effet/Ø, elle a un grand trou dans son manteau d’hiver.

Emilie ne prend visiblement pas soin de ses affaires.
En effet/Ø, elle a un grand trou dans son manteau d’hiver.

4.
Susanne avait l’impression qu’il lui manquait quelque chose.
Et en effet/Ø, elle a oublié son portefeuille dans le bus.

Susanne ne fait manifestement pas attention à son argent.
En effet/Ø, elle a oublié son portefeuille dans le bus.

5.
Les élèves craignaient que leur enseignant soit sévère.
Et en effet/Ø, il crie souvent très fort pour un rien.

Le maître d’école doit être un peu fatigué.
En effet/Ø, il crie souvent très fort pour un rien.

6.
Albert pensait qu’il serait puni pour avoir poussé sa sœur.
Et en effet/Ø, son père lui a confisqué son vélo pour un mois.

Albert a probablement fait une bêtise.
En effet/Ø, son père lui a confisqué son vélo pour un mois.

7.
Karine soupçonnait que son amie avait un amoureux secret.
Et en effet/Ø, elle vient de se marier avec un beau garçon.

Karine a une amie très chanceuse.
En effet/Ø, elle vient de se marier avec un beau garçon.

8.
Luc pensait que son travail était apprécié.
Et en effet/Ø, son directeur vient de le nommer chef d'équipe.

Luc est visiblement un excellent travailleur.
En effet/Ø, son directeur vient de le nommer chef d'équipe.

9 .
Franck pensait que ses voisins ne sortaient pas de la journée.
Et en effet/Ø, ils regardent des séries télévisées toute la journée.

Franck a des voisins manifestement très incultes.
En effet/Ø, ils regardent des séries télévisées toute la journée.

10.
Barbara pensait que sa sœur perdrait au tennis.
Et en effet/Ø, elle l'a battue une fois de plus en deux sets.

Barbara semble plus en forme physiquement que sa sœur.
En effet/Ø, elle l'a battue une fois de plus en deux sets.

11.
Rick avait l'impression d'avoir la tête qui tourne.
Et en effet/Ø, il est tombé en faisant du vélo ce matin.

Rick semble avoir un mauvais sens de l'équilibre.
En effet/Ø, il est tombé en faisant du vélo ce matin.

12.
Jean craignait que les sportifs soient des fêtards.
Et en effet/Ø, les footballeurs ont passé toute la nuit au pub.

Le match a dû être un succès.
En effet/Ø, les footballeurs ont passé toute la nuit au pub.

13.
Max s'imaginait que Matthieu n'avait pas de voiture.
Et en effet/Ø, il va travailler à vélo tous les jours.

Matthieu ne doit pas avoir son permis de conduire.
En effet/Ø, il va travailler à vélo tous les jours.

14.
Robert craignait de se faire mal en faisant du sport.
Et en effet/Ø, il s'est blessé en jouant au hockey.

Robert doit avoir très mal à la jambe.
En effet/Ø, il s'est blessé en jouant au hockey.

15.

Lise pensait qu’on avait cambriolé son appartement.
Et en effet/Ø, on a volé sa nouvelle télévision et ses bijoux.

La porte d’entrée de Lise a probablement été forcée.
En effet/Ø, on a volé sa nouvelle télévision et ses bijoux.

16.
Louis pensait que son cousin avait un poste d’ouvrier.
Et en effet/Ø, il travaille comme ouvrier du bâtiment depuis six mois.

Louis doit avoir de la force dans les bras.
En effet/Ø, il travaille comme ouvrier du bâtiment depuis six mois.

17.
Diane craignait de se faire renverser en faisant son jogging.
Et en effet/Ø, elle a été renversée par une voiture devant chez elle.

Diane ne devait pas porter de vêtements fluorescents.
En effet/Ø, elle a été renversée par une voiture devant chez elle.

18.
Jeanne s’attendait à être augmentée par son patron.
Et en effet/Ø, elle a reçu une grosse augmentation le mois passé.

Jeanne doit faire du bon travail.
En effet/Ø, elle a reçu une grosse augmentation le mois passé.

19.
Hélène s’attendait à avoir faim depuis qu’elle était au régime.
Et en effet/Ø, elle a déjà faim à 11 heures du matin.

Hélène a certainement oublié de prendre son petit déjeuner.
En effet/Ø, elle a déjà faim à 11 heures du matin.

20.
Jean craignait qu’on lui vole ses affaires pendant son voyage.
Et en effet/Ø, il s’est fait voler son vélo hier matin.

Jean n’a probablement pas de câble antivol.
En effet/Ø, il s’est fait voler son vélo hier matin.

21.
Tina pensait qu’elle pourrait avoir des problèmes en route.
Et en effet/Ø, sa voiture est tombée en panne sur l’autoroute.

Tina a probablement oublié de faire le plein.
En effet/Ø, sa voiture est tombée en panne sur l’autoroute.

22.

Les sapeurs-pompiers pensaient que l’incendie se propagerait rapidement.
Et en effet/Ø, le feu s’étend à une allure foudroyante.

Les sapeurs-pompiers ont dû arriver trop tard près du bâtiment.
En effet/Ø, le feu s’étend à une allure foudroyante.

23.
Marc pensait qu’Elise descendrait les marches trop vite.
Et en effet/Ø, elle a descendu les escaliers quatre à quatre.

Elise doit être très pressée ce matin.
En effet/Ø, elle a descendu les escaliers quatre à quatre.

024
Line espérait courir avec David.
Et en effet/Ø, ils ont fait la course ensemble hier soir.

Line et David semblent être de bons amis.
En effet/Ø, ils ont fait la course ensemble hier soir.

025
Martine craignait de faire naufrage.
Et en effet/Ø, les vagues ont fait chavirer le bateau tout de suite.

La mer doit être démontée.
En effet/Ø, les vagues ont fait chavirer le bateau tout de suite.

26.
Jacques craignait d’arriver en retard s’il prenait sa voiture.
Et en effet/Ø, il est arrivé cinq fois en retard au travail ce mois-ci.

Jacques doit avoir des problèmes personnels.
En effet/Ø, il est arrivé cinq fois en retard au travail ce mois-ci.

27.
Valérie espérait pouvoir déménager dans le Sud.
Et en effet/Ø, elle a déménagé en Espagne au printemps.

Valérie aime certainement le soleil et la chaleur.
En effet/Ø, elle a déménagé en Espagne au printemps.

28.
François avait l’impression d’avoir mal répondu aux questions.
Et en effet/Ø, il a encore raté son examen de linguistique ce semestre.

François n’est probablement pas un bon étudiant.
En effet/Ø, il a encore raté son examen de linguistique ce semestre.

29.
Anne pensait pouvoir aller skier prochainement.

Et en effet/Ø, elle est allée skier toute la journée lundi dernier.

Anne semble être en bonne condition physique.
En effet/Ø, elle est allée skier toute la journée lundi dernier.

30.
Jean pensait que Pierre avait des tableaux de peintres connus.
Et en effet/Ø, il a trois tableaux de Picasso dans son salon.

Pierre doit être très riche.
En effet/Ø, il a trois tableaux de Picasso dans son salon.

31.
Le ministre de l'agriculture avait des prévisions pessimistes pour les fermiers.
Et en effet/Ø, beaucoup de fermiers vont essuyer des pertes cette année.

La politique du ministre de l'agriculture ne semble pas porter ses fruits.
En effet/Ø, beaucoup de fermiers vont essuyer des pertes cette année.

32.
Sophie pensait que beaucoup de gens avaient un long trajet le matin.
Et en effet/Ø, de plus en plus de gens prennent le train pour aller travailler.

La compagnie des chemins de fer fournit certainement d'excellents services.
En effet/Ø, de plus en plus de gens prennent le train pour aller travailler.

33.
Sandra craignait que les alpinistes soient mal équipés.
Et en effet, ils n'ont pas pris le bon matériel avec eux.

Les alpinistes semblent être très inexpérimentés.
En effet/Ø, ils n'ont pas pris le bon matériel avec eux.

34.
Les politiciens étaient certains de gagner les élections.
Leur parti a encore gagné les élections ce mois-ci.

Les politiciens ont assurément mené une bonne campagne.
En effet/Ø, leur parti a encore gagné les élections ce mois-ci.

35.
Marie pensait que les tableaux de Serge auraient du succès.
Et en effet, ses tableaux se vendent partout dans le monde.

Ce peintre est vraisemblablement très à la mode.
En effet/Ø, ses tableaux se vendent partout dans le monde.

36.
Les soldats craignaient une attaque de leurs ennemis.
Et en effet/Ø, leurs ennemis sont arrivés par surprise depuis la ville voisine.

Les soldats étaient probablement mal préparés.
En effet/Ø, leurs ennemis sont arrivés par surprise depuis la ville voisine.

37.
Emilie pensait qu'elle aurait congé.
Et en effet/Ø, les cours sont annulés exceptionnellement demain matin.

La maîtresse doit être malade.
En effet/Ø, les cours sont annulés exceptionnellement demain matin.

38.
Léa pensait que le bébé était en train de faire ses dents.
Et en effet/Ø, ses premières dents sont en train de pousser ces jours-ci.

Le bébé doit avoir entre six et douze mois.
En effet/Ø, ses premières dents sont en train de pousser ces jours-ci.

039
Paul craignait que son téléphone soit de mauvaise qualité.
Et en effet/Ø, ses batteries sont toujours plates très vite.

Ce téléphone est visiblement de mauvaise qualité.
En effet/Ø, ses batteries sont toujours plates très vite.

40.
Max craignait de tomber malade.
Et en effet, il vient de s'enrhumer à nouveau.

Max semble avoir une santé fragile.
En effet/Ø, il vient de s'enrhumer à nouveau.

Appendix 2: Summaries of the fitted linear mixed effects models of each segment in Experiment 1

	SEGMENT 5			SEGMENT 6			SEGMENT 7		
	B	*CI*	*p*	*B*	*CI*		*B*	*CI*	
**Fixed Parts**									
(Intercept)	906.56	816.25 – 996.87	***<.001***	980.3	889.62 – 1070.97	***<.001***	999.46	895.67 – 1103.26	***<.001***
Connective (implicit vs. explicit)	55.75	18.43 – 93.07	***0.004***	-49.61	-85.48 – -13.73	***0.009***	20.53	-17.58 – 58.63	*0.291*
Relation (causal vs. confirmation)	-41.74	-82.64 – -0.85	***0.049***	-9.5	-45.51 – 26.50	*0.605*	4.94	-40.93 – 50.80	*0.834*
Group (L1-French vs. L2-French	269.77	112.92 – 426.63	***0.001***	343.64	177.09 – 510.19	***<.001***	281.71	89.47 – 473.96	***0.006***
Connective by relation	33.95	-28.60 – 96.51	*0.291*	-35.62	-106.23 – 34.98	*0.323*	-19.1	-94.98 – 56.78	*0.622*
Connective by group	8.1	-66.41 – 82.62	*0.832*	-56.22	-127.86 – 15.42	*0.13*	-68.85	-144.95 – 7.25	*0.076*
Relation by group	-35.85	-98.20 – 26.51	*0.263*	-12.1	-82.62 – 58.41	*0.737*	-34.07	-109.80 – 41.66	*0.378*
Connective by Relation by group	60.23	-64.67 – 185.13	*0.347*	23.62	-117.54 – 164.79	*0.743*	-160.81	-312.44 – -9.18	***0.038***
**Random Parts**									
σ^2	144415.508			184854.21			213164.359		
$\tau_{00,\ participant}$	90714.014			101714.856			136370.878		
$\tau_{00,\ item}$	20871.002			13399.434			15967.219		
ρ_{01}	0.17			-0.631			1		
$N_{participant}$	59			59			59		
N_{item}	40			40			40		
$ICC_{participant}$	0.354			0.339			0.373		
ICC_{item}	0.082			0.045			0.044		
Observations	2293			2293			2293		
R^2 / Ω_0^2	.505 / .503			.457 / .455			.470 / .469		

Appendix 3: Summary of the fitted linear mixed effects model in Experiment 2

	COHERENCE		
	B	*CI*	*p*
**Fixed Parts**			
(Intercept)	3.87	3.60 – 4.15	***<.001***
Connective (implicit vs. explicit)	0.03	-0.10 – 0.16	*0.658*
Relation (causal vs. confirmation)	-0.27	-0.61 – 0.06	*0.188*
Group (L1-French vs. L2-French	0.02	-0.20 – 0.24	*0.847*
Connective by relation	-0.07	-0.33 – 0.19	*0.593*
Connective by group	0.53	0.27 – 0.79	***<.001***
Relation by group	0.5	0.24 – 0.75	***<.001***
Connective by Relation by group	0.04	-0.48 – 0.55	*0.888*
**Random Parts**			
σ^2	1.247		
$\tau_{00, \text{participant}}$	0.117		
$\tau_{00, \text{item}}$	0.084		
ρ_{01}	1		
$N_{\text{participant}}$	58		
N_{item}	5		
$ICC_{\text{participant}}$	0.081		
ICC_{item}	0.058		
Observations	1154		
R^2 / Ω_0^2	.211 / .204		
